# Increased serum soluble PD-l1 levels in patients with advanced stages of chronic kidney disease

**DOI:** 10.3389/fmed.2025.1530804

**Published:** 2025-01-31

**Authors:** Ayaka Hayashi, Hiroto Ishihara, Mayuko Kawabe, Kazuhiko Kato, Akio Nakashima, Izumi Yamamoto, Teppei Sakano, Hiroe Kobashi, Makoto Morita, Takashi Yokoo, Mitsuyoshi Urashima

**Affiliations:** ^1^Division of Molecular Epidemiology, The Jikei University School of Medicine, Tokyo, Japan; ^2^Division of Nephrology and Hypertension, Department of Internal Medicine, The Jikei University School of Medicine, Tokyo, Japan; ^3^Division of Innovation for Medical Information Technology, The Jikei University School of Medicine, Tokyo, Japan; ^4^Allm, Inc., Tokyo, Japan; ^5^Department of Infectious Disease, Team Medical Clinic, Tokyo, Japan; ^6^Pfizer Japan Inc., Tokyo, Japan

**Keywords:** PD-1, PD-L1, chronic kidney disease, dialysis, immune checkpoint inhibitor, soluble PD-L1 (sPD-L1)

## Abstract

**Background:**

Programed death-ligand 1 (PD-L1) is overexpressed on renal tubular and vascular epithelial cells in inflammatory kidney diseases as well as on aged kidney podocytes, contributing to chronic kidney disease (CKD) progression. The association of serum soluble programed death-ligand 1 (sPD-L1) levels and chronic kidney disease (CKD) progression is unknown.

**Methods:**

To compare serum sPD-L1 levels among healthy individuals and patients with various CKD stages, including those undergoing dialysis, a secondary analysis was performed using clinical data and residual serum samples from four distinct cohorts, each prospectively collected for different research purposes: The Vaccine Cohort (2021–2022), the Cancer Cohort (2010–2018), the Dialysis Initiation Cohort (2023–2024), and the Dialysis Maintenance Cohort (2011–2015) included patients on stable maintenance dialysis.

**Results:**

The study analyzed serum sPD-L1 levels in 2,829 participants (mean age, 54.2 years; male, 54.2%) across the four cohorts. In the Vaccine and Cancer cohorts, sPD-L1 levels increased significantly with age (*P* < 0.001) and male sex (*P* < 0.001). In the Vaccine Cohort, elevated median sPD-L1 levels (pg/mL) were significantly associated with CKD stage progression (*P* < 0.001), showing exponentially higher levels with CKD progression. A similar association was observed and validated in the Cancer Cohort (*P* < 0.001). In the Dialysis Initiation Cohort (*n* = 15), sPD-L1 levels significantly increased three months after dialysis initiation compared to pre-dialysis levels (*P* = 0.03). In the Dialysis Maintenance Cohort, sPD-L1 levels increased with longer dialysis duration (*P* < 0.001).

**Conclusion:**

Serum sPD-L1 levels might increase with CKD stage progression, dialysis initiation and longer dialysis duration. Further clinical investigation is required to confirm these results.

## Introduction

As the world’s elderly population has grown, the number of patients with chronic kidney disease (CKD) has increased in recent decades. Multiple factors, such as kidney aging, glomerulonephritis and comorbidities, influence the progression of CKD in the elderly. While many innovative therapies for CKD have been developed, the mechanism of kidney aging remains unclear and its understanding has recently become an emerging topic.

In kidneys, aging leads to progressive nephron loss and increased glomerulosclerosis. Podocyte senescence plays a key role in this age-related glomerulosclerosis: Recent studies have shown that podocyte loss is associated with kidney aging (1) and that overexpression of programed cell death-1 (PD-1) in aged podocytes leads to cell death by interacting with its ligand, programed cell death ligand 1 (PD-L1), resulting in podocyte loss (2). As immune checkpoint molecules, PD-1 and PD-L1 play crucial roles in maintaining immune homeostasis. A representative example of this interaction is the blockade of anti-tumor immunity; PD-L1 on the membrane of cancer cells suppresses anti-tumor immunity by binding to the PD-1 receptor primarily on activated T cells (3). Previous studies have shown that in inflammatory kidney diseases where T cells play a prominent role in immune responses, such as allograft rejections, glomerulonephritis and tubulointerstitial nephritis, PD-L1 is overexpressed on renal tubular and vascular epithelial cells, suggesting the possibility of its renoprotective function (4–6). In kidney aging, the interaction between PD-1 and PD-L1 has recently drown great attention. Pippin et al has demonstrated overexpression of PD-1 in aged murine and human podocytes, which correlated with lower eGFR, higher segmental glomerulosclerosis, and vascular damage (2, 7). In turn, blocking PD-1 with an antibody inhibited apoptosis of podocytes and improved phenotype aging in murine kidneys (2). This activation of PD-1 signaling needs its ligand PD-L1, but it has not been determined how PD-L1 initiates the signaling in podocytes. Similar to its membrane-bound form, soluble PD-L1 (sPD-L1) in the blood is thought to exert inhibitory effects on immune function by binding to PD-1 on T cells (8, 9), and elevated blood levels of sPD-L1 are reportedly associated with various conditions, such as cancer (10, 11), aging (12), pregnancy (13), and diseases (8), e.g., acute coronary syndrome (14), severe viral infections, e.g., COVID-19 (15), and severe sepsis (16).

It is possible that the circulating sPD-L1 also affects the PD-1 signaling in podocytes. Thus, aging is hypothesized to elevate sPD-L1 levels in blood, as well as PD-1 expression on podocytes, potentially facilitating podocyte death via interaction between these molecules, thereby advancing CKD stages.

It is important to have an understanding of the relationship between serum sPD-L1 levels and kidney function before proceeding with detailed investigations, which is the aim of this study.

## Materials and methods

### Study design and cohort

**FIGURE 1:**
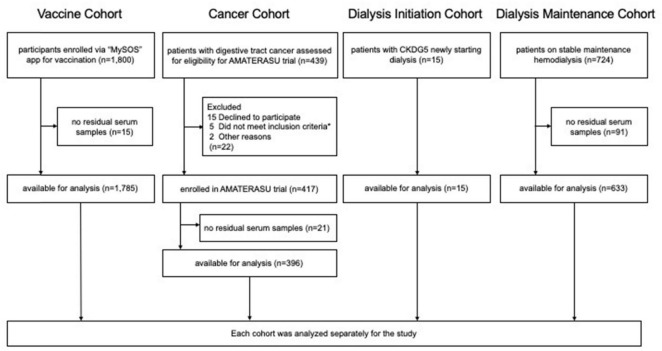
Flowchart of the four cohorts. * (1) a histopathological diagnosis of epithelial carcinoma of the digestive tract (esophagus, stomach, small intestine, colon, and rectum) clinical stages I to III, (2) aged 30 to 90 years at entry, (3) diagnosis and initial surgery at the International University of Health and Welfare Hospital, (4) not taking vitamin D supplements or active vitamin D, and (5) no history of urinary tract stones.

This secondary study utilized clinical data and residual serum samples from four previously conducted clinical studies: the Vaccine, Cancer, Dialysis Initiation, and Dialysis Maintenance cohorts. Participants provided informed consent, either in a written or electronic form, allowing their clinical information and samples to be used in subsequent studies, with the option to opt out of *post hoc* analyses. All procedures were conducted in accordance with the Declaration of Helsinki. The flowchart of the cohort selection is shown in Figure 1.

#### Vaccine Cohort

This prospective cohort study investigated factors influencing SARS-CoV-2 antibody titers. A total of 1,800 volunteers aged ≥ 16 years were enrolled via the “MySOS” health app in Japan from December 2021 to November 2022, regardless of comorbidities. The study protocol was approved by the Tentakai Clinical Research and Ethics Committee (2020-08[001]). Their medical information was collected through application questionnaires, and baseline serum samples were obtained at the time of enrollment before vaccination. Of the 1,800 participants, 1,785 were tested for sPD-L1 using residual baseline serum samples and enrolled in this study as the Vaccine Cohort.

#### Cancer Cohort

This cohort included 417 patients aged ≥ 30 years with digestive tract cancer, enrolled in the AMATERASU randomized, double-blind, placebo-controlled trial at the International University of Health and Welfare Hospital, Tochigi, Japan, from January 2010 to February 2018 (UMIN000001977) (17). The inclusion criteria for the AMATERASU trial were: (1) a histopathological diagnosis of epithelial carcinoma of the digestive tract (esophagus, stomach, small intestine, colon, and rectum) clinical stages I to III, (2) aged 30–90 years at entry, (3) diagnosis and initial surgery at the International University of Health and Welfare Hospital, (4) not taking vitamin D supplements or active vitamin D, and (5) no history of urinary tract stones. The exclusion criteria included: (1) tumors that were not resectable by surgery, (2) serious postoperative complications at enrollment, (3) pathological diagnosis other than epithelial carcinoma (such as malignant lymphoma and sarcoma), and (4) pathological stage 0 or IV. The study protocol was approved by the ethics committees of both the International University of Health and Welfare Hospital (FK-1) and Jikei University School of Medicine (21–216 [6094]). Of the 417 patients enrolled in the AMATERASU trial, 396 were tested for sPD-L1 using residual serum samples and enrolled in this study as the Cancer Cohort.

#### Dialysis Initiation Cohort

This cohort included 15 patients who began hemodialysis or peritoneal dialysis at Jikei University Hospital between December 2023 and April 2024. Serum sPD-L1 levels were measured at the start of dialysis (pre) and three months later (post). The study protocol was approved by the Jikei University Institutional Review Board (33-097 [10712]). All patients were enrolled as the Dialysis Initiation Cohort.

#### Dialysis Maintenance Cohort

This prospective cohort study enrolled 724 hemodialysis patients aged ≥ 20 years from 15 outpatient units in Tokyo, Japan, between May 2011 and June 2015 (18). All patients had been on dialysis for at least 3 months and received thrice-weekly sessions lasting 3 to 5 hours each. Patients with unstable conditions such as having acute bleeding, active infection, and acute coronary syndrome at baseline were excluded at the time of enrollment. The protocol was approved by the Jikei University Institutional Review Board (22-182 [6359]). Of the 724 patients, 633 were tested for sPD-L1 using residual serum samples and enrolled in this study as the Dialysis Maintenance Cohort.

### Data collection

Clinical data (age, sex, body mass index, medical history, comorbidities, baseline laboratory tests, dialysis details, etc.) were obtained from the original databases. Serum samples were stored at −80°C until use.

### Measurement of sPD-L1 levels

Serum sPD-L1 levels were measured in undiluted serum samples using the Human PD-L1 ELISA Kit [28-8] (ab277712, Abcam, UK) according to the manufacturer’s instructions, with a detection range of 3.9–1,300 pg/mL. The lot number was consistent within each cohort, but differed among the four cohorts. Consequently, sPD-L1 levels were compared within the same cohort, but not between different cohorts, except when sPD-L1 levels were compared between individuals treated with and without dialysis. sPD-L1 levels were analyzed as continuous variables and were divided into quintiles and tertiles.

### Formula for estimated glomerular filtration rate and CKD classification

Serum creatinine (Cr) levels were obtained from the medical records of patients in the cancer, dialysis maintenance and dialysis initiation cohorts, and creatinine was measured in stored samples in the Vaccine Cohort. eGFR was calculated using the Japanese creatinine-based formula (JPN-Cre), and CKD stages were classified according to KDIGO guidelines (eGFR < 60 mL/min/1.73 m^2^) (19).

### Statistical analysis

Categorical variables were compared using chi-squared tests, while continuous variables were analyzed using unpaired t-tests, Mann-Whitney U tests, or regression analysis, as appropriate. The Wilcoxon matched-pairs signed-rank test was used to compare sPD-L1 levels before and after dialysis initiation in the same patients. Survival analysis was conducted in the Dialysis Maintenance Cohort, and was performed with cardiovascular disease or all-cause death as the primary outcome, and infections requiring hospitalization or all-cause death as the secondary outcomes. Hazard ratios (HRs) and 95% confidence intervals (CIs) were determined for these outcomes using a Cox proportional hazards model. Statistical significance was set at *P* < 0.05. All analyses were performed using Stata 18.0 (StataCorp LLC., College Station, TX, United States).

## Results

### Participants’ characteristics

Patient’s baseline characteristics are detailed in Table 1. The study included a total of 2,829 participants with measurable residual serum sPD-L1 levels (mean age, 54.2 years; male, 54.2%): 1,785 from the Vaccine Cohort (99.2% of the original sample), 396 (96.1%) from the Cancer Cohort, 15 (100%) from the Dialysis Initiation Cohort, and 633 (53.2%) from the Dialysis Maintenance Cohort. The Vaccine Cohort included a younger population with fewer men and fewer comorbidities than the other three cohorts. In the Dialysis Maintenance Cohort, all of whom were on hemodialysis, median dialysis vintage was 80 (38–148) months, and median Kt/V was 1.4 (1.2–1.5). In the Dialysis Initiation Cohort, there were 13 (86.7%) hemodialysis and two (13.3%) peritoneal dialysis cases. Blood access types were: 12 arteriovenous fistulas (80.0%), two (13.3%) peritoneal dialysis catheters, and one (6.7%) central venous catheter. Baseline urine output was maintained at a median volume of 1631 ± 946 mL/day.

**TABLE 1:** Baseline characteristics of patients in the four cohorts.

Characteristic	Vaccine Cohort (*n* = 1785)	Cancer Cohort (*n* = 396)	Dialysis Initiation Cohort (*n* = 15)	Dialysis Maintenance Cohort (*n* = 633)
Age, years	49 (40–57)	66.2 ± 10.6	63.4 ± 14.9	65 (56–72)
Sex, male, n (%)	807 (45.3)	265 (66.9)	12 (80.0)	447 (70.6)
Body mass index, kg/m^2^	22.8 (20.6–25.6)	21.9 (19.8–23.8)	23.1	21.4 (19.3–24.3)
**Comorbidity, n (%)**				
Hypertension	247 (13.9)	155 (39.1)	15 (100)	413 (66.6)
Diabetes	64 (3.6)	65 (16.4)	8 (53.3)	242 (38.2)
Dyslipidemia	198 (11.1)	175 (44.9)	–	182 (28.8)
Acute coronary syndrome	9 (0.5)	18 (4.6)	0 (0)	–
Stroke	3 (0.2)	16 (4.0)	1 (6.7)	–
Cardiovascular disease*	12 (0.7)	30 (7.6)	1 (6.7)	121 (19.1)
Cancer	25 (1.4)	15 (3.8)	3 (20.0)	11 (1.7)
Serum Cr, mg/dL	0.73 (0.63–0.86)	0.75 (0.64–0.87)	9.2 ± 2.6	11.7 ± 3.0
eGFR, mL/min/1.73 m^2^	76.1 (67.2–86.8)	74.1 (63.6–83.0)	5.3 ± 1.4	3.7 (3.2–4.5)
**CKD stage, n (%)**				
Stage 1	342 (19.2)	153 (13.4)	0 (0)	0 (0)
Stage 2	1272 (71.4)	273 (68.9)	0 (0)	0 (0)
Stage 3a	154 (8.6)	54 (13.6)	0 (0)	0 (0)
Stage 3b	10 (0.6)	16 (4.0)	0 (0)	0 (0)
Stage 4	0 (0)	0 (0)	0 (0)	0 (0)
Stage 5	2 (0.1)	0 (0)	0 (0)	0 (0)
Stage 5d	2 (0.1)	0 (0)	15 (100)	633 (100)

*Cardiovascular disease includes acute coronary syndrome and stroke. eGFR, estimated glomerular filtration rate; CKD, chronic kidney disease. Values are presented as the n (%), mean ± standard deviation, or median (IQR, 25th to 75th percentiles).

### Age, sex, and sPD-L1 levels

**FIGURE 2:**
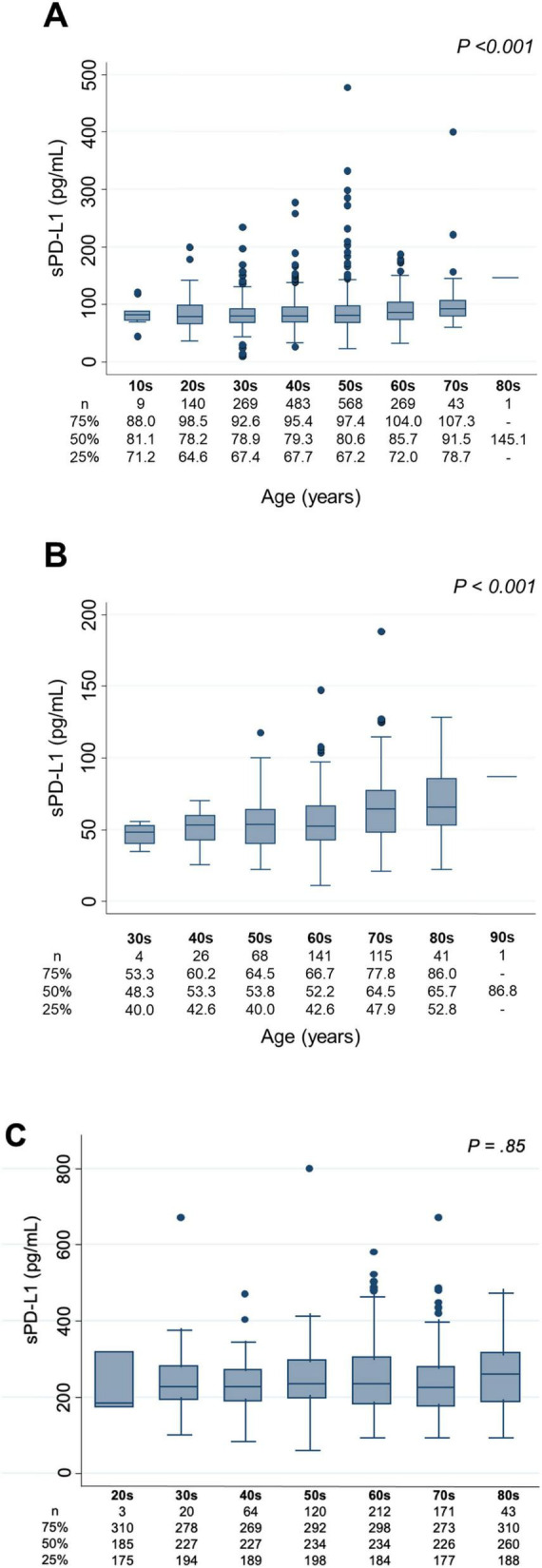
Association between age in decades and sPD-L1 levels. **(A)** Vaccine Cohort. **(B)** Cancer Cohort. **(C)** Dialysis maintenance cohort. sPD-L1 levels by age group with box plots: The number of participants and the median sPD-L1 level with interquartile range of each age group are presented below.

**FIGURE 3:**
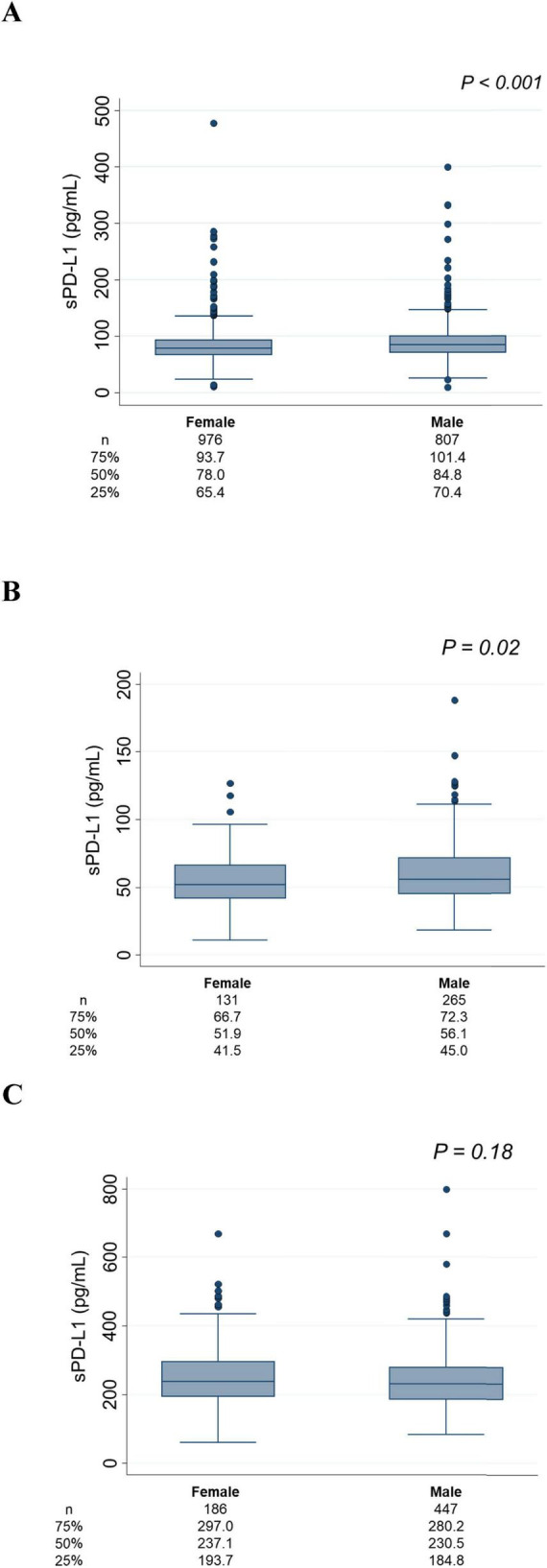
Association between sex and sPD-L1 levels. **(A)** Vaccine Cohort. **(B)** Cancer Cohort. **(C)** Dialysis maintenance cohort. The number of patients and the median sPD-L1 level with interquartile range of each group are shown below.

Associations between age and sPD-L1 levels are presented in Figure 2. In the Vaccine Cohort, sPD-L1 levels significantly increased with age (*P* < 0.001) (Figure 2A), with a similar positive association in the Cancer Cohort (*P* < 0.001) (Figure 2B). This association between age and sPD-L1 levels in the Vaccine and Cancer cohorts remained significant after adjusting for sex and kidney function (Vaccine Cohort: *P* < 0.001, Cancer Cohort: *P* < 0.001), but not significant in the Dialysis Maintenance Cohort (Figure 2C). Notably, there was a substantial variation in sPD-L1 levels even among individuals of the same age group. Furthermore, in the Vaccine Cohort, median [IQR] sPD-L1 levels were significantly higher in males than females (84.8 [70.4–101.4] and 78.0 [65.4–93.7] pg/mL, respectively, *P* < 0.001) (Figure 3A). This association between sex and sPD-L1 levels remained significant even after adjusting for age (*P* < 0.001). Similar sex-related differences were observed in the Cancer Cohort (Figure 3B), but not in the Dialysis Maintenance Cohort (Figure 3C).

### sPD-L1 levels and CKD stages

**FIGURE 4:**
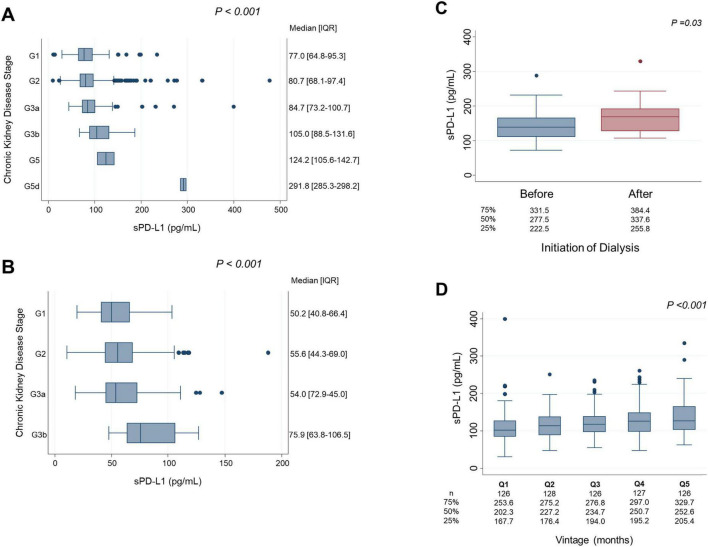
sPD-L1 levels and CKD stages. **(A)** Vaccine Cohort: Linear regression analysis was performed to examine the association between sPD-L1 levels and CKD stage, adjusted for age and sex. **(B)** Cancer Cohort: Linear regression analysis was used to assess the relationship between sPD-L1 levels and CKD stage, adjusted for age and sex. **(C)** Dialysis initiation cohort: The Wilcoxon matched-pairs signed-rank test was used to compare sPD-L1 levels before and after dialysis initiation in the same patients. The median sPD-L1 levels with interquartile range of before and after dialysis initiation are shown below. **(D)** Dialysis maintenance cohort: Linear regression analysis was conducted to evaluate the association between quintiles of dialysis duration and sPD-L1 levels, adjusted for age and sex. The number of patients and the median sPD-L1 level with interquartile range of each quintile are shown below.

Figure 4 illustrates the association between serum sPD-L1 levels and CKD stages. In the Vaccine Cohort, higher sPD-L1 levels were significantly associated with advancing CKD stage (*P* < 0.001), even after adjusting for age, sex, hypertension, diabetes, and cancer (β 0.031, 95% CI 0.019–0.043, *P* < 0.01) (Table 2). Median levels of sPD-L1 (pg/mL) showed an exponential increase with CKD stage progression, with levels from 77.0 (G1) and 80.7 (G2) in individuals without CKD, 84.7 (G3a), 105.0 (G3b), and 124.2 (G5) in patients with CKD, to 291.8 (G5d) in those undergoing dialysis (Figure 4A). A similar pattern was observed in the Cancer Cohort (*P* < 0.001) (Figure 4B). The association remained significant after adjusting for hypertension, diabetes, and cardiovascular disease, along with age and sex (β 0.033, 95% CI 0.0074–0.059, *P* = 0.012) (Table 3). In the Dialysis Initiation Cohort, sPD-L1 levels before and after dialysis initiation in the same patients were compared, sPD-L1 levels increased significantly, from 277.5 pg/mL just before dialysis to 337.6 pg/mL three months after dialysis initiation (*P* = 0.03) (Figure 4C). In the Dialysis Maintenance Cohort, longer dialysis duration was associated with higher sPD-L1 levels (*P* < 0.001) (Figure 4D). This difference remained significant after adjusting hypertension, diabetes, cardiovascular disease, cancer, and Kt/V, along with age and sex (β 0.00030, 95% CI 0.00000016–0.000044, *P* = 0.001) (Table 4).

**TABLE 2:** Factors associated with soluble PD-L1 levels* by univariate and multivariate linear regression models in Vaccine Cohort.

Variables	Univariate Coeff. (95% CI)	*p*-value	Model 1		Model 2	
			**Multivariate** **Coeff. (95% CI)**	***p*-value**	**Multivariate** **Coeff. (95% CI)**	***p*-value**
Age	0.0013 (0.00081 to 0.0019)	<0.001	0.00033 (−0.00026 to 0.00092)	0.276	–	–
Sex, male	0.034 (0.021 to 0.046)	<0.001	0.029 (0.017 to 0.042)	<0.001	–	–
Hypertension	0.039 (0.020 to 0.57)	<0.001	0.020 (0.00095 to 0.039)	0.040	0.027 (0.0080 to 0.045)	0.005
Diabetes mellitus	0.0034 (0.00017 to 0.068)	0.049	0.011 (−0.024 to 0.045)	0.544	0.018 (−0.016 to 0.053)	0.292
Acute coronary syndrome	−0.0081 (−0.097 to 0.81)	0.858	–	–	–	–
Stroke	−0.035 (−0.19 to 0.12)	0.647	–	–	–	–
Cardiovascular disease**	−0.015 (−0.092 to 0.062)	0.700	–	–	–	–
Cancer	0.076 (0.022 to 0.13)	0.006	0.069 (0.017 to 0.12)	0.010	0.063 (0.010 to 0.12)	0.019
CKD stage	0.038 (0.27 to 0.048)	<0.001	0.031 (0.019 to 0.043)	<0.001	0.035 (0.024 to 0.046)	<0.001

*Soluble PD-L1 and eGFR were transformed to an ordinary logarithm (log10).

**Cardiovascular disease includes acute coronary syndrome and stroke. PD-L1, programmed death ligand 1; CVD, cardiovascular disease; CKD, chronic kidney disease.

**TABLE 3:** Factors associated with soluble PD-L1 levels* by univariate and multivariate linear regression models in Cancer Cohort.

Variable	Univariate Coeff. (95% CI)	*p*-value	Model 1		Model 2		Model 3	
			**Multivariate Coeff.** **(95% CI)**	***p*-value**	**Multivariate Coeff.** **(95% CI)**	***p*-value**	**Multivariate Coeff.** **(95% CI)**	***p*-value**
Age	0.0040 (0.0025 to 0.0055)	<0.001	0.0032 (0.0016 to 0.0048)	<0.001	0.0032 (0.0016 to 0.0048)	<0.001	–	–
Sex, male	0.049 (0.014 to 0.084)	0.006	0.046 (0.012 to 0.079)	0.008	0.046 (0.012 to 0.080)	0.008	–	–
Hypertension	0.031 (−0.0029 to 0.065)	0.073	–	–	−0.0057 (−0.041 to 0.030)	0.752	0.0067 (−0.029 to 0.043)	0.716
Diabetes mellitus	0.036 (−0.0092 to 0.080)	0.119	–	–	0.025 (−0.020 to 0.069)	0.277	0.0243 (−0.023 to 0.069)	0.320
Acute coronary syndrome	0.088 (0.0088 to 0.17)	0.029	–	–	–	–	–	–
Stroke	0.083 (−0.0010 to 0.17)	0.053	–	–	–	–	–	–
Cardiovascular disease	0.073 (0.011 to 0.14)	0.022	0.044 (−0.017 to 0.10)	0.157	0.043 (−0.019 to 0.10)	0.170	0.055 (−0.0077 to 0.12)	0.085
CKD stage	0.053 (0.028 to 0.078)	<0.001	0.033 (0.0071 to 0.059)	0.012	0.033 (0.0074 to 0.059)	0.012	0.048 (0.023 to 0.074)	<0.001

*Soluble PD-L1 was transformed to an ordinary logarithm (log10).

**Cardiovascular disease includes acute coronary syndrome and stroke. PD-L1, programed death ligand 1; CKD, chronic kidney disease.

**TABLE 4:** Factors associated with soluble PD-L1 levels* by univariate and multivariate linear regression models in dialysis maintenance cohort.

Variables	Univariate Coeff. (95% CI)	*p*-value	Multivariate Coeff. (95% CI)	*p*-value
Age	0.0016 (0.00048 to 0.0027)	0.005	−0.00022 (−0.0012 to 0.00081)	0.677
Sex, male	−0.0050 (−0.035 to 0.025)	0.744	−0.0099 (−0.037 to 0.017)	0.472
Hypertension	−0.017 (−0.047 to 0.012)	0.251	−0.017 (−0.043 to 0.0084)	0.189
Diabetes mellitus	−0.024 (−0.052 to 0.0044)	0.097	0.0041 (−0.026 to 0.027)	0.976
Cardiovascular disease	0.015 (−0.020 to 0.050)	0.409	0.011 (−0.020 to 0.042)	0.506
Cancer	0.015 (−0.016 to 0.046)	0.342	0.00030 (−0.020 to 0.044)	0.468
HD vintage	0.031 (0.0010 to 0.060)	0.043	0.00030 (0.000016 to 0.000044)	<0.001
Kt/V	0.078 (0.069 to 0.088)	<0.001	0.033 (−0.014 to 0.079)	0.166

*Soluble PD-L1 and eGFR were transformed to an ordinary logarithm (log10).

**Cardiovascular disease includes acute coronary syndrome and stroke. PD-L1, programed death ligand 1; HD, hemodialysis.

### sPD-L1 levels between dialysis-treated patients and non-dialysis patients

**FIGURE 5:**
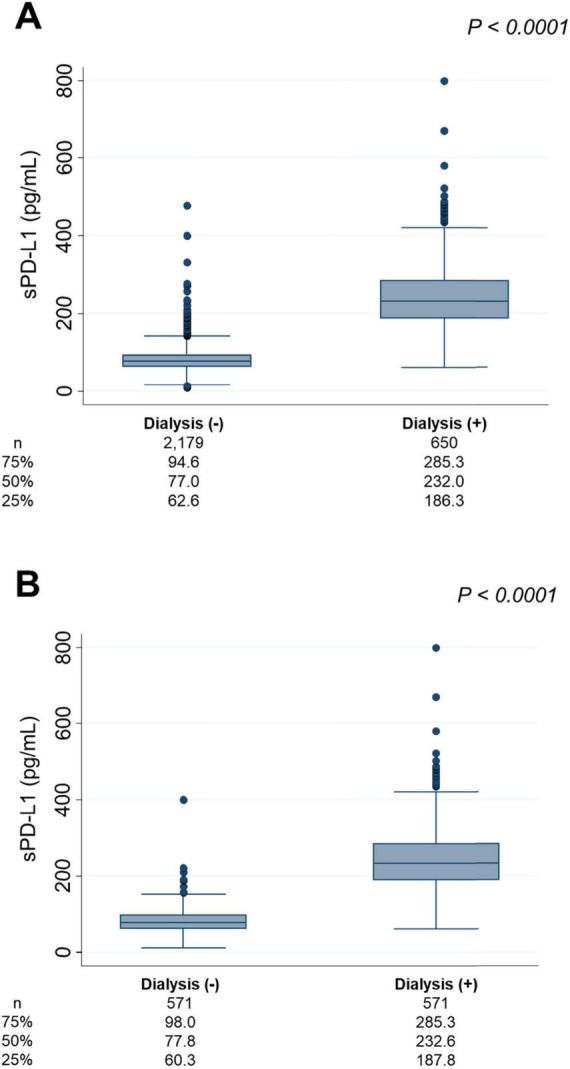
sPD-L1 levels and dialysis. **(A)** Comparison of sPD-L1 levels between dialysis patients and non-dialysis individuals. **(B)** Comparison of sPD-L1 levels between dialysis patients and non-dialysis individuals in a 1:1 ratio, matched for age and sex. The number of patients and the median sPD-L1 level with interquartile range of each group are shown below.

Across the four cohorts, 650 patients were either undergoing dialysis or about to initiate dialysis at the time of sPD-L1 measurement, while 2,179 individuals were either CKD patients without dialysis or non-CKD individuals (CKD stage G1-G5). The median [IQR] sPD-L1 level in dialysis patients was 232.0 [186.3–285.3] pg/mL, which was significantly higher than that in non-dialysis CKD patients or non-CKD individuals, whose median level was 77.0 [62.6–94.6] pg/mL (*P* < 0.0001), representing a three-fold increase in dialysis patients (Figure 5A). This difference remained nearly unchanged after 1:1 matching for age and sex (Figure 5B).

## Discussion

In this study, serum sPD-L1 levels were found to increase exponentially with CKD stage progression, independent of age, sex, or other confounding factors, a finding further validated by assessing another cohort. Although numerous studies have evaluated sPD-L1, few have described its association with kidney function. sPD-L1 is produced by either alternative splicing or proteolytic cleavage of membrane PD-L1, and has a molecular weight of approximately 37–56 kDa (8). sPD-L1 might be excreted in urine (20, 21). Thus, serum sPD-L1 levels increase with reduction of GFR, as evidenced by the fact that sPD-L1 levels increased with CKD progression in the present study.

Furthermore, median sPD-L1 levels were found to be three times higher in dialysis patients compared to non-dialysis individuals. To determine whether this significant difference was attributable to the dialysis procedure itself, sPD-L1 levels were compared before and after the initiation of dialysis. Evaluation revealed an increase of approximately 100 pg/mL in sPD-L1 levels three months after starting dialysis, suggesting that dialysis contributes, at least in part, to the elevation of serum sPD-L1 levels. Additionally, sPD-L1 levels were directly related to dialysis duration. The molecular weight cutoff for molecules filtered by low-flux and high-flux hemodialysis membranes typically ranges from 5 to 15 kDa and 50 to 60 kDa, respectively. Considering its molecular weight, i.e., 37–56 kDa (8), it is likely that sPD-L1 accumulates in the blood, as it is not effectively filtered by low-flux dialysis, which was used in most patients in the Dialysis Initiation and Maintenance cohorts in this study. In patients on dialysis, urine volume progressively decreases as dialysis vintage increases (22). It is hypothesized that initiation of dialysis reduces urine volume, leading to a decrease in the amount of sPD-L1 excreted in urine, and hence, its elevation. Platelet activation (23) and vascular endothelial cell damage or stimulation (8) caused by hemodialysis procedure itself or an arteriovenous fistula might also have contributed to the observed elevation in serum sPD-L1 levels in dialysis patients in this study. Previous studies have suggested that sPD-L1 levels are higher in patients with coronary artery disease than in those without (8), and that elevated sPD-L1 levels, induced during the development of atherosclerosis, are associated with future cardiovascular events (24). These findings suggest that sPD-L1 levels might reflect the severity and instability of atherosclerotic lesions as well.

While CKD typically advances with aging, there are cases where its progression outpaces what would be expected based solely on chronological age. The drivers of this accelerated decline are complex and multifactorial, including cellular senescence, inflammation, mitochondrial dysfunction, disruptions in sirtuin and Klotho signaling, and dysregulation of the autophagy-lysosome system (25). These overlapping mechanisms make it difficult to pinpoint a singular cause. Nevertheless, Pippin et al. demonstrated that PD-1 expression on podocytes increases with age, a phenomenon closely tied to renal functional decline and worsening glomerulosclerosis due to podocyte apoptosis caused by PD-1/PD-L1 pathway upregulation (2). Interestingly, blocking PD-1 with an antibody has been shown to reduce both podocyte senescence and inflammation, thus extending the lifespan of these critical cells (2). In the present study, sPD-L1 levels increased significantly with age, independent of sex and kidney function, and also exhibited significant variability even within the same age and sex group. Contrary to the previous hypothesis, elevated sPD-L1 in blood, beyond what is expected with aging, might stimulate PD-1 on podocytes, thereby impairing renal function and advancing CKD progression. However, PD-L1 overexpression can also occur with aging podocytes, inflamed renal tubular epithelial cells (26), renal interstitial tissues involved in fibrosis development (27), and kidney function decline itself, suggesting that these additional factors should be carefully considered in future clinical investigations. Moreover, a recent meta-analysis of randomized clinical trials demonstrated that combined use of anti-PD-1 or PD-L1 antibodies with chemotherapy increased the risk of severe acute kidney injury and the composite of all severe acute kidney adverse events (28, 29). Therefore, blocking the interaction between PD-L1 and PD-1 with antibody therapy might not necessarily halt age-related CKD progression, and could potentially worsen it, probably through autoimmune mechanisms.

## Limitations

This study has several limitations. First, each cohort was primarily enrolled for different study purposes, resulting in notable differences in baseline characteristics. The Vaccine Cohort consisted of relatively healthy individuals with few comorbidities, the Cancer Cohort included patients with esophageal, gastric, or colorectal cancer, and the Dialysis Initiation and Dialysis Maintenance Cohorts comprised patients who had newly started dialysis or were on stable dialysis, respectively. Second, in the Vaccine and Cancer Cohorts, since creatinine and sPD-L1 levels were measured from the same blood samples, it was not possible to determine whether elevated sPD-L1 levels led to renal functional decline or vice versa. Additionally, since an association does not necessarily imply a causal relationship, while patients with elevated sPD-L1 levels in this study exhibited advanced CKD stages, caution is needed when interpreting these findings. Third, although a significant increase in sPD-L1 levels was observed after the initiation of dialysis, the Dialysis Initiation Cohort included only 15 participants, which was fewer than in other cohorts, raising the possibility that this could be a chance finding. Fourth, since PD-1 on podocytes in the renal glomeruli were not evaluated pathologically, it remains unclear whether the interaction between circulating sPD-L1 and PD-1 on podocytes accelerated kidney aging and contributed to CKD stage progression. Fifth, when measuring sPD-L1 levels across the four cohorts, ELISA kit lot numbers were consistent within each cohort, but differed between cohorts. Hence, it was not possible to make subtle comparisons between different cohorts. For example, while dialysis patients had levels three times higher than non-dialysis patients, with the lot number differences falling within the margin of error, in cases where the differences were smaller, such as between cancer and non-cancer patients, it was necessary to avoid combining patient data across cohorts.

## Conclusion

To the best of our knowledge, this is the first study demonstrating the association between serum sPD-L1 levels and CKD progression, with sPD-L1 reaching the highest levels in patients with stage 5 CKD. Serum sPD-L1 levels also increased with dialysis initiation and dialysis duration. Further investigation is needed to understand the mechanism of sPD-L1 elevation and its impact on patients with CKD.

## Data Availability

The data analyzed in this study is subject to the following licenses/restrictions: The principal investigator had full access to all the data in the study and takes responsibility for the integrity of the data and the accuracy of the data analysis. Requests to access these datasets should be directed to MU, urashima@jikei.ac.jp.

## References

[1] Shankland SJ Rule AD Kutz JN Pippin JW Wessely O. Podocyte senescence and aging. *Kidney360.* (2023) 4:1784–93. 10.34067/KID.0000000000000284 37950369 PMC10758523

[2] Pippin JW Kaverina N Wang Y Eng DG Zeng Y Tran U Upregulated PD-1 signaling antagonizes glomerular health in aged kidneys and disease. *J Clin Invest.* (2022) 132:e156250. 10.1172/JCI156250 35968783 PMC9374384

[3] Boussiotis VA. Molecular and biochemical aspects of the PD-1 checkpoint pathway. *N Engl J Med.* (2016) 375:1767–78. 10.1056/NEJMra1514296 27806234 PMC5575761

[4] Schoop R Wahl P Le Hir M Heemann U Wang M Wüthrich RP. Suppressed T-cell activation by IFN-gamma-induced expression of PD-L1 on renal tubular epithelial cells. *Nephrol Dial Transplant.* (2004) 19:2713–20. 10.1093/ndt/gfh423 15353579

[5] Chen Y Zhang J Li J Zou L Zhao T Tang Y Expression of B7-H1 in inflammatory renal tubular epithelial cells. *Nephron Exp Nephrol.* (2006) 102:e81–92. 10.1159/000089686 16282703

[6] Starke A Lindenmeyer MT Segerer S Neusser MA Rüsi B Schmid DM Renal tubular PD-L1 (CD274) suppresses alloreactive human T-cell responses. *Kidney Int.* (2010) 78:38–47. 10.1038/ki.2010.97 20393451

[7] Wang Y Eng DG Kaverina NV Loretz CJ Koirala A Akilesh S Global transcriptomic changes occur in aged mouse podocytes. *Kidney Int.* (2020) 98:1160–73. 10.1016/j.kint.2020.05.052 32592814 PMC7606654

[8] Bailly C Thuru X Quesnel B. Soluble programmed death ligand-1 (sPD-L1): a pool of circulating proteins implicated in health and diseases. *Cancers (Basel).* (2021) 13:3034. 10.3390/cancers13123034 34204509 PMC8233757

[9] Niu M Liu Y Yi M Jiao D Wu K. Biological characteristics and clinical significance of soluble PD-1/PD-L1 and Exosomal PD-L1 in cancer. *Front Immunol.* (2022) 13:827921. 10.3389/fimmu.2022.827921 35386715 PMC8977417

[10] Cui Q Li W Wang D Wang S Yu J. Prognostic significance of blood-based PD-L1 analysis in patients with non-small cell lung cancer undergoing immune checkpoint inhibitor therapy: a systematic review and meta-analysis. *World J Surg Oncol.* (2023) 21:318. 10.1186/s12957-023-03215-2 37821941 PMC10566159

[11] Sun J Hu S Li X. Meta-analysis of the prognostic value of soluble programmed death ligand-1 (sPD-L1) in cancers. *Biomarkers.* (2023) 28:477–85. 10.1080/1354750X.2023.2198168 37017446

[12] Chen Y Wang Q Shi B Xu P Hu Z Bai L Development of a sandwich ELISA for evaluating soluble PD-L1 (CD274) in human sera of different ages as well as supernatants of PD-L1+ cell lines. *Cytokine.* (2011) 56:231–8. 10.1016/j.cyto.2011.06.004 21733718

[13] Okuyama M Mezawa H Kawai T Urashima M. Elevated soluble PD-L1 in pregnant women’s serum suppresses the immune reaction. *Front Immunol.* (2019) 10:86. 10.3389/fimmu.2019.00086 30833943 PMC6387906

[14] Fujisue K Yamamoto E Sueta D Takae M Nishihara T Komorita T Increased soluble programed cell death-ligand 1 is associated with acute coronary syndrome. *Int J Cardiol.* (2022) 349:1–6. 10.1016/j.ijcard.2021.11.060 34843822

[15] Akhmaltdinova L Mekhantseva I Turgunova L Kostinov M Zhumadilova Z Turmukhambetova A. Association of soluble PD-L1 and NLR combination with 1-Year mortality in patients with COVID-19. *Int Immunopharmacol.* (2024) 129:111600. 10.1016/j.intimp.2024.111600 38325048

[16] Zhao Y Jia Y Li C Shao R Fang Y. Predictive value of soluble programmed death-1 for severe sepsis and septic shock during the first week in an intensive care unit. *Shock.* (2019) 51:289–97. 10.1097/SHK.0000000000001171 29702526

[17] Urashima M Ohdaira H Akutsu T Okada S Yoshida M Kitajima M Effect of vitamin D supplementation on relapse-free survival among patients with digestive tract cancers: the AMATERASU randomized clinical trial. *JAMA.* (2019) 321:1361–9. 10.1001/jama.2019.2210 30964526 PMC6459116

[18] Nakashima A Ohkido I Yokoyama K Mafune A Urashima M Yokoo T. Proton pump inhibitor use and magnesium concentrations in hemodialysis patients: a cross-sectional study. *PLoS One.* (2015) 10:e0143656. 10.1371/journal.pone.0143656 26618538 PMC4664382

[19] Kidney Disease: Improving Global Outcomes (KDIGO) CKD Work Group. KDIGO 2024 clinical practice guideline for the evaluation and management of chronic kidney disease. *Kidney Int.* (2024) 105:S117–314. 38490803 10.1016/j.kint.2023.10.018

[20] Xuan Y Chen F Qin L He R Yuan J. sPD-1 and sPD-L1 levels in serum and urine of patients with primary nephrotic syndrome and their clinical significance. *Clin Lab* (2021) 67. 10.7754/Clin.Lab.2020.200913 34655201

[21] Lv Y Gai K Ding X Sun W. Soluble forms of PD-1 and sPD-L1/2 in serum and urine of patients with head and neck cancer and their clinical significance. *Biotechnol Genet Eng Rev.* (2024) 40:2234–45. 10.1080/02648725.2023.2199237 37057626

[22] Lee MJ Park JT Park KS Kwon YE Oh HJ Yoo TH Prognostic value of residual urine volume, GFR by 24-hour urine collection, and eGFR in patients receiving dialysis. *Clin J Am Soc Nephrol.* (2017) 12:426–34. 10.2215/CJN.05520516 28228465 PMC5338702

[23] Li Y Xin G Li S Dong Y Zhu Y Yu X PD-L1 regulates platelet activation and thrombosis via caspase-3/GSDME pathway. *Front Pharmacol.* (2022) 13:921414. 10.3389/fphar.2022.921414 35784685 PMC9240427

[24] Miyazaki S Fujisue K Yamanaga K Sueta D Usuku H Tabata N Prognostic significance of soluble PD-L1 on cardiovascular outcomes in patients with coronary artery disease. *J Atheroscler Thromb.* (2024) 31:355–67. 10.5551/jat.64183 37793811 PMC10999719

[25] Yamamoto T Isaka Y. Pathological mechanisms of kidney disease in ageing. *Nat Rev Nephrol.* (2024) 20:603–15. 10.1038/s41581-024-00868-4 39025993

[26] Ding H Wu X Gao W. PD-L1 is expressed by human renal tubular epithelial cells and suppresses T cell cytokine synthesis. *Clin Immunol.* (2005) 115:184–91. 10.1016/j.clim.2005.01.005 15885642

[27] Zhang Y Mi X Zhang Y Li J Qin Y He P Immune checkpoint activity exacerbate renal interstitial fibrosis progression by enhancing PD-L1 expression in renal tubular epithelial cells. *Transl Res* (2024) 271:52–67. 10.1016/j.trsl.2024.05.004 38723861

[28] Trisal SR Low G Pathan F Gangadharan Komala M. Kidney adverse events associated with immune checkpoint inhibitor therapy: a systematic review and Bayesian network meta-analysis. *Clin J Am Soc Nephrol.* (2023) 18:843–9. 10.2215/CJN.0000000000000160 36999976 PMC10356161

[29] Righini M Mollica V Rizzo A La Manna G Massari F. Renal toxicities in cancer patients receiving immune-checkpoint inhibitors: a meta-analysis. *J Clin Med.* (2022) 11:4373. 10.3390/jcm11154373 35955989 PMC9368813

